# Association of secondhand smoke exposure with allergic multimorbidity in Korean adolescents

**DOI:** 10.1038/s41598-020-73430-4

**Published:** 2020-10-02

**Authors:** Ahnna Lee, Sook Young Lee, Kang-Sook Lee

**Affiliations:** 1 Department of Public Health, Graduate School, The Catholic University of Korea, Seoul, Korea; 2 Department of Preventive Medicine, College of Medicine, The Catholic University of Korea, Seoul, Korea; 3 Division of Pulmonary, Allergy and Critical Care Medicine, Department of Internal Medicine, College of Medicine, Seoul St. Mary’s Hospital, The Catholic University of Korea, Seoul, Korea

**Keywords:** Diseases, Risk factors, Epidemiology

## Abstract

This study aimed to examine the health effect of secondhand smoke (SHS) exposure at home, school, and/or public places on allergic multimorbidity using nationwide data among school-attending adolescents in Korea. Allergic multimorbidity was defined as two or more coexisting allergic diagnoses of asthma, allergic rhinitis, and/or atopic dermatitis during the past 12 months. A multinomial logistic regression analysis was performed to evaluate the association of SHS exposure and allergic multimorbidity. Of the study participants, 24.3% were diagnosed as having any allergic disease currently and 66.3% reported SHS exposure. Any SHS exposure that includes public places conferred increased odds of atopic dermatitis in non-current smokers (adjusted odds ratio 1.21–1.46; 95% confidence interval [CI] 1.10–1.66). Moreover, when controlling for current smoking additionally, SHS exposure at the three sites was 1.37 and 1.96 times more likely to be associated with allergic single and multiple morbidities, respectively (95% CI 1.26–1.49 and 1.65–2.31, respectively). In conclusion, this study found positive associations of SHS exposure with single or multiple allergic morbidity compared to no exposure at all. Further studies with longitudinal designs and objective measurement of SHS exposure and allergic diagnosis are warranted.

## Introduction

The prevalence of allergic disease has been steadily increasing worldwide. A large-scale research project^1^ that covered > 50 countries has shown increases in the prevalence of allergic diseases, including asthma, allergic rhinitis, and atopic dermatitis, among children in many countries. In Korea, the prevalence of allergic diseases among adolescents were reported to be 7.2%, 35.3%, and 22.5% for asthma, allergic rhinitis, and atopic dermatitis, respectively, in 2019^2^. The increase in the overall prevalence of allergic diseases has led to increased economic costs in Korea, with allergic rhinitis being the third most costly disease among 500 medical conditions in 2018. According to the statistics of the National Health Insurance Corporation, the medical costs for the treatment of asthma, allergic rhinitis, and atopic dermatitis in 2018 were 261.8, 509.3, and 81.6 billion won, respectively^3^.

Links between allergic diseases have been suggested because they share common immunopathological mechanisms. Asthma and allergic rhinitis, or atopic dermatitis and airway allergic diseases often coexist with each other^4–6^. Epidemiological studies^7^ have suggested that food allergy may be associated with the progression of atopic disorders described as “atopic march”. Cutaneous manifestations such as atopic dermatitis proceed with subsequent migration of sensitized T cells into the upper and lower airways, resulting in systemic allergic responses such as asthma and allergic rhinitis^8^. Multimorbidity, the occurrence of two or more diseases in an individual, is known to increase disease severity and duration, healthcare costs, length of hospital admission, and mortality rate^9,10^. Moreover, allergic morbidity diminishes patient quality of life and impairs physical function^11^.

According to the US Surgeon General’s report, no amount of exposure to secondhand smoke(SHS) is “safe”^12^. SHS is a mixture of smoke emitted from the burning tip of tobacco products and the smoke exhaled by the smoker^13^. When inhaled, sidestream smoke becomes more toxic and carcinogenic than mainstream smoke, causing damage to the respiratory epithelium and sensory irritation^14^. The global public health treaty, that is, the framework convention on tobacco control (FCTC), provided guidelines for the implementation of article 8 (protection from exposure to tobacco smoke) to enforce a 100% smoke-free environment^13^. However, among US students, 55.9% still reported SHS exposure in at least 1 location among home, school, and indoor/outdoor public area^15^.

SHS exposure is known to be one of the most common risk factors of allergic morbidity. An animal study^16^ showed elevated granulocyte–macrophage colony-stimulating factor and interleukins 2 and 5 levels in ovalbumin and tobacco smoke-exposed group, which suggests that environmental tobacco smoke (ETS) can be related to allergic sensitization. Another adolescent murine model^17^ demonstrated that exposure to tobacco smoke and house dust mite concomitantly results in T-helper cell type 2-associated asthmatic phenotype. Cigarette smoke exposure even for short-term facilitated allergic sensitization and subsequent asthma development. Meanwhile, the onset of allergic disease during childhood and adolescence can develop into a chronic disease, requiring great caution^18^.

To date, however, studies regarding SHS exposure have focused more on single allergic diseases, not on allergic multimorbidity. Moreover, in the literature, the place of exposure to tobacco smoke in children has been limited mostly to homes. Considering that school-aged children spend a substantial amount of time not only at home but also outside the home, exposure location may play an important role in the development of multiple allergic diseases. Therefore, an epidemiological analysis to examine the impact of SHS exposure at various locations on allergic multimorbidity is needed. This study aimed to analyze the association of SHS exposure at home, school, and/or public places with childhood development of asthma, allergic rhinitis, and/or atopic dermatitis among Korean adolescents.

## Results

### Secondhand smoke exposure according to socioeconomic variables

The total study population consisted of 55,748 students who were attending middle and high schools in Korea. Table 1 presents the estimated prevalence of SHS exposure according to the socioeconomic characteristics of the Korean adolescent population. Overall, > 6 in 10 students (66.3%) were exposed to ETS in Korea. Self-reported low- and middle-low-income households were more prevalent in the SHS-exposed group than in the non-exposed group (2.1–11.4% vs. 1.8–7.9%). Meanwhile, self-reported middle-to-high- and high-income households were more prevalent in the non-exposed group (29.5% and 13.9%, respectively) than in the SHS-exposed group (9.6% and 28.3%, respectively).

**Table 1:** Second hand smoke exposure at home, school, and/or public places according to socioeconomic factors (n = 55,748).

	Total participants n = 55,748(100)	No SHS exposure n = 18,960(33.7)	SHS exposure n = 36,788(66.3)	*p* value
**Residential area**				.691
Metropolitan cities	24,658 (42.5)	8425 (42.7)	16,233 (42.5)	
Small and medium sized cities	26,738 (51.9)	9004 (51.6)	17,734 (52.1)	
Rural	4352 (5.5)	1531 (5.7)	2821 (5.5)	
**Household income**				< .001*
High	6104 (11.0)	2605 (13.9)	3499 (9.6)	
High-Middle	15,794 (28.7)	5559 (29.5)	10,235 (28.3)	
Middle	26,856 (48.0)	8908 (46.9)	17,948 (48.6)	
Middle-Low	5811 (10.2)	1528 (7.9)	4283 (11.4)	
Low	1183 (2.0)	360 (1.8)	823 (2.1)	
**Paternal education**				< .001*
High school or below	9063 (15.8)	2524 (13.0)	6539 (17.2)	
College or above	19,642 (36.3)	6428 (34.7)	13,214 (37.1)	
Unknown	25,552 (45.5)	9527 (50.0)	16,025 (43.2)	
Father absence	1491 (2.5)	481 (2.3)	1010 (2.5)	
**Maternal education**				< .001*
High school or below	10,369 (18.4)	2921 (15.4)	7448 (20.0)	
College or above	19,035 (34.8)	6224 (33.3)	12,811 (35.6)	
Unknown	25,001 (44.6)	9436 (49.4)	15,565 (42.1)	
Mather absence	1343 (2.2)	379 (1.3)	964 (2.4)	

*SHS* secondhand smoke exposure.

*Significance at *p* < 0.05.

### General characteristics of the study population with allergic diseases

Among the study participants, 24.3% were diagnosed by a physician as having current allergic disease (Table 2). Girls were more likely to have an allergic disease than boys (52% vs. 48%). Allergic diseases were more prevalent in children who smoked. Specifically, the overall weighted prevalence of current smoking was 7.8% among the allergic children, which was significantly higher than the 3.7% among the non-exposed children (*p* < 0.001). Table 3 presents the allergic morbidity rates based on SHS exposure at different locations. All the allergic participants were more likely to be exposed to SHS at home, school, and public places concurrently than the non-allergic participants (8.5–22.9% vs. 7.2%).

**Table 2:** General characteristics of the study population with lifetime and current allergic disease (n = 55,748).

	Total participants	Allergic participants			
		Lifetime	*p* value	Current	*p* value
	n = 55,748(100)	n = 26,310(47.8)		n = 13,255(24.3)	
**Sex**			< .001*		< .001*
Boys	29,059 (52.0)	12,865 (48.7)		6406 (48.0)	
Girls	26,689 (48.0)	13,445 (51.3)		6849 (52.0)	
**Grade**			< .001*		< .001*
Middle school	28,675 (48.1)	12,993 (46.1)		6518 (46.1)	
High school	27,073 (51.9)	13,317 (53.9)		6737 (53.9)	
**Residential area**			< .001*		< .001*
Metropolitan cities	24,658 (42.5)	11,856 (43.0)		5938 (42.4)	
Small and medium sized cities	26,738 (51.9)	12,660 (48.0)		6511 (53.2)	
Rural	4352 (5.5)	1794 (4.9)		806 (4.4)	
**Household income**			< .001*		
High	6104 (11.0)	2718 (10.4)		1420 (10.8)	< .001*
High-Middle	15,794 (28.7)	7685 (29.6)		4048 (30.9)	
Middle	26,856 (48.0)	12,542 (47.5)		6090 (45.7)	
Middle-Low	5811 (10.2)	2811 (10.5)		1380 (10.3)	
Low	1183 (2.0)	554 (2.0)		317 (2.3)	
**Obesity**			< .001*		.013
Underweight	3187 (5.9)	1728 (6.7)		854 (6.5)	
Normal	40,379 (72.6)	18,952 (72.1)		9533 (72.1)	
Overweight	5597 (10.0)	2609 (10.0)		1307 (9.8)	
Obese	6585 (11.6)	3021 (11.3)		1561 (11.5)	
**Vigorous physical activity**			< .001*		.476
No	47,223 (85.3)	18,029 (69.6)		8941 (68.4)	
Yes	8525 (14.7)	8281 (30.4)		4314 (31.6)	
**Current smoking**			.471		< .001*
No	53,789 (96.3)	24,532 (93.1)		12,245 (92.2)	
Yes	1959 (3.7)	1778 (6.9)		1010 (7.8)	

*Significance at *p* < 0.05.

**Table 3:** Allergic multimorbidity (Asthma, Allergic rhinitis, and/or Atopic dermatitis) according to the location of secondhand smoke exposure (n = 55,748).

SHS exposure	Current no morbidity	Current allergic morbidity, n(%)						
		Single morbidity			Multiple morbidity (2 +)			
		AS	AR	AD	AS and AR	AS and AD	AR and AD	AS, AR and AD
SHS exposure at home, school, and public place	2989 (7.2)	25 (9.0)	740 (8.5)	187 (8.8)	42 (12.5)	11 (22.9)	144 (11.0)	25 (17.7)
SHS exposure at home and school	840 (1.9)	9 (2.8)	193 (2.0)	44 (2.4)	5 (1.5)	2 (3.4)	28 (2.0)	2 (1.0)
SHS exposure at home and public place	5441 (12.6)	40 (13.9)	1186 (12.9)	316 (14.8)	49 (12.6)	5 (13.2)	203 (14.8)	20 (10.5)
SHS exposure at school and public place	3380 (8.3)	15 (5.6)	921 (10.7)	174 (8.8)	38 (10.8)	2 (4.6)	145 (11.5)	18 (12.6)
SHS exposure at home only	4072 (9.1)	28 (8.8)	764 (8.2)	195 (9.0)	32 (8.5)	3 (9.0)	95 (7.2)	6 (3.6)
SHS exposure at school only	1214 (2.8)	5 (2.2)	276 (3.1)	47 (2.0)	12 (4.0)	–	33 (2.4)	7 (5.2)
SHS exposure at public place only	9541 (22.9)	63 (24.9)	2235 (24.9)	497 (24.8)	76 (20.1)	7 (15.1)	317 (25.2)	24 (17.7)
No exposure	15,016 (35.0)	86 (32.7)	2706 (29.6)	622 (29.5)	119 (29.9)	14 (31.8)	347 (25.9)	50 (31.6)

*SHS* secondhand smoke exposure, *AS* asthma, *AR* allergic rhinitis, *AD* atopic dermatitis.

*Significance at *p* < 0.05.

### Association between exposure to secondhand smoke and atopic dermatitis

We found a significant association between exposure to SHS and atopic dermatitis among both current and non-current smokers (Table 4). Current-smoking children exposed to tobacco smoke at home, school, and public places concurrently were more likely to have been diagnosed as having atopic dermatitis than children without SHS exposure (adjusted odds ratio [aOR] 1.52; 95% confidence interval [CI] 1.08–2.15). Likewise, SHS exposure at the three sites was 1.46 times more likely to report atopic dermatitis in non-current smokers (95% CI 1.28–1.66). Any SHS exposure that includes public places was related to increased odds of atopic dermatitis in non-current smokers (aOR 1.21–1.46; 95% CI 1.10–1.66).

**Table 4:** Multiple logistic regression model of the association of secondhand smoke exposure with diagnosis of current atopic dermatitis by current smoking (n = 55,748).

SHS exposure	Current-smoker (n = 3758)			Non-current smoker (n = 51,990)		
	Atopic dermatitis			Atopic dermatitis		
	n (%)	Crude	Adjusted*	n(%)	Crude	Adjusted*
		OR (95% CI)			OR(95% CI)	
Total	306 (100)			3284 (100)		
SHS exposure at home, school, and public place	75 (23.2)	**1.61 (1.14, 2.28)**	**1.52 (1.08, 2.15)**	292 (8.9)	**1.57 (1.38, 1.77)**	**1.46 (1.28, 1.66)**
SHS exposure at home and school	10 (4.1)	**2.68 (1.33, 5.37)**	**2.60 (1.32, 5.12)**	66 (2.0)	1.23 (0.95, 1.59)	1.15 (0.90, 1.49)
SHS exposure at home and public place	38 (12.4)	0.95 (0.63, 1.43)	0.87 (0.57, 1.32)	506 (14.8)	**1.42 (1.27, 1.59)**	**1.31 (1.17, 1.47)**
SHS exposure at school and public place	36 (12.3)	1.13 (0.78, 1.65)	1.06 (0.72, 1.55)	303 (9.7)	**1.37 (1.19, 1.57)**	**1.27 (1.10, 1.45)**
SHS exposure at home only	19 (5.8)	0.86 (0.54, 1.39)	0.82 (0.51, 1.33)	280 (8.4)	1.11 (0.96, 1.27)	1.06 (0.92, 1.22)
SHS exposure at school only	10 (3.2)	1.33 (0.67, 2.64)	1.33 (0.67, 2.67)	77 (2.1)	0.88 (0.68, 1.14)	0.84 (0.65, 1.09)
SHS exposure at public place only	54 (18.9)	1.04 (0.72, 1.50)	0.97 (0.67, 1.41)	791 (25.1)	**1.29 (1.17, 1.42)**	**1.21 (1.10, 1.34)**
No exposure	64 (20.0)	1	1	969 (29.1)	1	1

Bold values denote statistical significance at the *p* < 0.05 level.

*SHS* secondhand smoke exposure.

*Adjusted for sex, school grade, obesity, residential area, household income, paternal and maternal education.

### Association between exposure to secondhand smoke and allergic multimorbidity

Table 5 shows the relationship between SHS exposure and allergic multimorbidity. After adjusting for confounding variables, the participating students who had been exposed to tobacco smoke at home, school, and public places concurrently reported the highest odds of single allergic morbidity (aOR 1.37; 95% CI 1.26–1.49). Any exposure location that includes public places (i.e., SHS exposure at public places only, at home and public places, at school and public places, and at home, school, and public places) were significantly associated with allergic multimorbidity with aORs of 1.23 (95% CI 1.08–1.41), 1.33 (1.15–1.53), 1.59 (1.35–1.88), and 1.96 (1.65–2.31).

**Table 5:** Multinomial logistic regression model of the association of secondhand smoke exposure with current allergic multimorbidity (n = 55,748).

SHS exposure	Single morbidity (1)			Multiple morbidity (2 +)		
	n(%)	Crude	Adjusted	n(%)	Crude	Adjusted
		OR(95% CI)*			OR(95% CI)*	
Total	11,374 (100)			1881 (100)		
SHS exposure at home, school, and public place	952 (8.6)	**1.41 (1.30, 1.53)**	**1.37 (1.26, 1.49)**	222 (12.1)	**2.16 (1.84, 2.54)**	**1.96 (1.65, 2.31)**
SHS exposure at home and school	246 (2.1)	**1.29 (1.10, 1.51)**	**1.25 (1.06, 1.46)**	37 (1.9)	1.23 (0.89, 1.72)	1.16 (0.83, 1.61)
SHS exposure at home and public place	1542 (13.2)	**1.24 (1.16, 1.32)**	**1.20 (1.12, 1.29)**	277 (14.0)	**1.43 (1.24, 1.64)**	**1.33 (1.15, 1.53)**
SHS exposure at school and public place	1110 (10.3)	**1.46 (1.34, 1.58)**	**1.36 (1.25, 1.48)**	203 (11.3)	**1.74 (1.48, 2.06)**	**1.59 (1.35, 1.88)**
SHS exposure at home only	987 (8.4)	1.08 (1.00, 1.17)	1.08 (1.00, 1.18)	136 (7.3)	1.02 (0.85, 1.23)	0.99 (0.82, 1.20)
SHS exposure at school only	328 (2.9)	**1.21 (1.06, 1.38)**	**1.16 (1.02, 1.33)**	52 (2.9)	1.29 (0.97, 1.72)	1.24 (0.93, 1.65)
SHS exposure at public place only	2795 (24.9)	**1.28 (1.21, 1.36)**	**1.22 (1.15, 1.29)**	424 (23.3)	**1.31 (1.15, 1.49)**	**1.23 (1.08, 1.41)**
No exposure	3414 (29.7)	1	1	530 (27.3)	1	1

Bold values denote statistical significance at the *p* < 0.05 level.

*SHS* secondhand smoke exposure.

*Adjusted for sex, school grade, obesity, residential area, household income, paternal and maternal education, and current smoking.

## Discussion

Our study confirms the positive association between exposure to secondhand tobacco smoke and allergic morbidity. First, we revealed that SHS exposure at home, school, and public places altogether have a significant connection with self-reported diagnosis of atopic dermatitis. Next, we also found that concurrent exposure to SHS at home, school, and public places is related to self-reported single allergic disease. Lastly, the likelihood of allergic multimorbidity was higher in the students exposed to SHS at smoke-free indoor public places than in the non-exposed students.

Our study shows that concurrent SHS exposure at home, school, and public places is associated significantly with atopic dermatitis. Passive smoking has been identified as a risk factor of atopic dermatitis in the literature^19^. ETS may act as irritants of the integumentary system, enabling potential allergens to be penetrate and causing eczema symptoms to manifest. The results of our previous study^20^ among adolescents also indicated that SHS exposure at home and school for ≥ 5 days per week was more likely to be associated with current atopic dermatitis with aORs of 1.42 (95% CI 1.18–1.40) and 1.42 (1.24–1.62), respectively. Local cutaneous sensitization in atopic dermatitis, manifested as a beginning of the ‘atopic march’, is often followed by subsequent development of systemic allergic response^8^.

The association of SHS exposure and allergic morbidity has been previously studied, but mostly as a single entity. A comprehensive analysis^21^ of tobacco smoke and allergic rhinitis, allergic dermatitis, and food allergy demonstrated that passive smoking among children and adolescents had an impact on allergic rhinitis (increased relative risk: 1.09; 95% CI 1.04–1.14). A meta-analysis^22^ of epidemiologic studies revealed that household passive smoke exposure increased the risk of incidence of asthma among children aged 5 to 18 years (OR 1.30, 95% CI 1.04–1.62). In a recent US national survey^23^, children with SHS exposure were 30% more likely to be diagnosed as having asthma than non-exposed children. When exposed to SHS for > 1 h for 7 days, asthmatic children showed an elevated risk of reporting symptoms such as wheezing, dry couth, and sleep disturbance^24^. In line with previous studies, our study revealed that SHS exposures at home, school, and public places are related to the highest odds of single allergic morbidity, which suggests that increased exposure sources may result in stronger association with symptoms.

The notable results of our study indicated that SHS exposure location anywhere that includes public places are all significantly associated with increased odds of allergic multimorbidity. Under the FCTC guidelines^13^, parties are encouraged to legislate complete smoking ban in indoor public places, indoor workplaces, and public transit. The Korean government has improved its tobacco control policies regarding the smoke-free legislation since the enactment of the National Health Promotion Act in 1995. It has gradually expanded to cover educational facilities, government buildings, hospitals, restaurants, and bars^25^. However, still designated smoking areas are accessible in public areas but are known to be ineffective in reducing SHS^13^. Our findings may be related to the partial adoption of the smoke-free law or low adherence rate to existing smoke-free regulations in public places.

In the present study, 66.3% of the sampled participants were exposed to SHS at home, school, and/or public place. An Indian tobacco survey^26^ revealed that SHS exposure was still reported by 27.0% and 39.3% of the overall respondents in 2016–2017 after the legislation of the smoke-free law in public transportations and restaurants. Poor compliance to the smoking ban legislation may negatively affect the improvement of health outcomes. In the literature, implementation of the tobacco control policies recommended by the World Health Organization has benefitted child health in terms of, for example, asthma exacerbation, respiratory tract infection, and reduced smoking-related mortality^27,28^. Deploying enforcement officers and imposing violation penalties for continuous monitoring, awareness-raising campaigns, and signage display have been suggested as successful policy enforcement measures^29^.

### Limitations

We examined a range of SHS exposure locations and allergic multimorbidities in a nationally representative population. The large sample size and high response rate of the survey participants, with which an acceptable representativeness was attained, are the notable strengths of this study. However, our study has several methodological limitations. First, the causal interpretation of the results was limited owing to the cross-sectional nature of the study design. Second, results based on self-reported data can be misreported as compared with studies that use objective biological markers such as urine or serum cotinine levels. Questioning regarding the days of exposure to SHS in those with allergic conditions may lead to an answer of higher number of days as they may be more sensitive to the effects of SHS and are more likely to report it by exaggerating. Therefore, the positive association between SHS exposure and allergic morbidity may be subject to recall bias. Lastly, the survey questionnaire was limited to tobacco smoke exposure from conventional cigarette smoking. Recent studies have revealed that secondhand e-cigarette aerosol exposure is associated with asthma attack among youth in Florida^30^. Future studies should consider aerosol or smoke from newly developed nicotine-containing products such as e-cigarettes or heated tobacco products.

## Conclusion

In conclusion, this study presents that SHS exposure is associated with single or multiple allergic morbidity among the youth. Additional studies with longitudinal designs and objective measurement of SHS exposure and allergic diagnosis are required to further evaluate the links between SHS exposure and allergic morbidity.

## Methods

### Data and study participants

**Figure 1:**
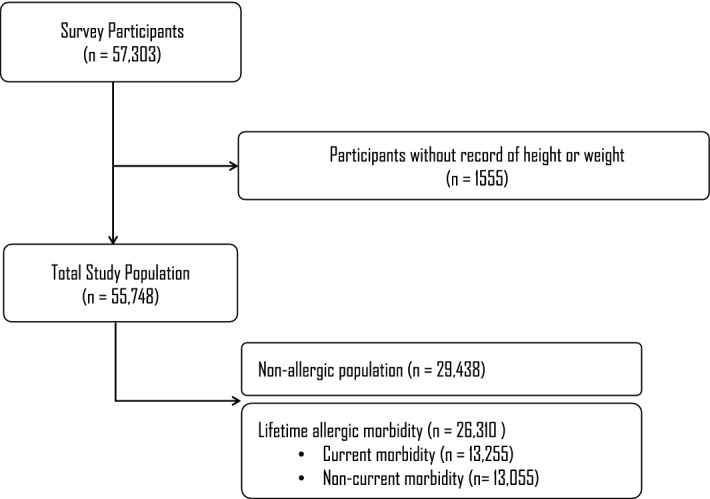
A schematic illustration of the participant selection process.

We collected data from the 15th Korea Youth Risk Behavior Survey (KYRBS)^31^, a nationwide population-based survey of health-risk behaviors among Korean adolescents. The KYRBS has been conducted by the Korea Centers for Disease and Prevention to the evaluate health statuses of students attending middle and high schools, using an anonymously self-administered questionnaire. It applies a complex sampling design involving stratification, clustering, and multistage sampling. The primary stage included the selection of 800 middle and high schools (400 each) from 117 strata according to administrative region and school type. The secondary unit included the selection of one class per grade from each sampled middle and high school. Among the selected 60,100 students, a nationwide representative sample of the 2,683,547 students from 5,611 schools in Korea, 57,303 participated in the survey, with a response rate of 95.3%. We excluded from the analysis participants who had no records of body weight and height. Finally, 55,748 school-attending students were included in the study (Fig. 1). Informed online written consent was obtained from all subjects prior to participation in the survey conducted in computer rooms. Parental consent was exempted because the survey was conducted in schools with great number of participants. This study protocol received approval from the Institutional Review Board of Catholic University of Korea (MC20ZASI0030). All study methods were performed in compliance with relevant guidelines and regulations.

### Secondhand smoke exposure (SHS)

SHS exposure at home was defined based on the participant’s answer to the question, “During the past 7 days, how many days were you exposed to tobacco smoke generated by someone else in your house?”. SHS exposure at school was defined based on the participant’s answer to the question, “During the past 7 days, how many days were you exposed to tobacco smoke generated by someone else at smoke-free indoor places (classroom, bathroom, corridor, etc.) in your school?”. SHS exposure at public places was defined based on the participant’s answer to the question, “During the past 7 days, how many days were you exposed to tobacco smoke generated by someone else at smoke-free indoor places (stores, restaurants, shopping mall, concert hall, internet cafe, karaoke, etc.) besides your home or school?”. The responses ranged from “0 day” to “7 days per week”. This variable was then classified as yes (1 day from 7 days per week) or no (0 day per week). Variables regarding exposure to SHS were classified as one of the following for analysis: SHS exposure at home only; at school only; at public places only; at home and school; at home and public places; at school and public places; and at home, school, and public places.

### Allergic disease

Lifetime allergic morbidity (asthma, allergic rhinitis, or atopic dermatitis) was evaluated using the question, “Have you ever been diagnosed with asthma/allergic rhinitis/atopic dermatitis by a physician in your lifetime?” (yes/no). Current allergic morbidity (asthma, allergic rhinitis, or atopic dermatitis) was evaluated using the question, “During the past 12 months, have you ever been diagnosed with asthma/allergic rhinitis/atopic dermatitis by a physician?” If the participant responded “yes,” we classified this as current asthma/allergic rhinitis/atopic dermatitis. Single allergic morbidity was defined as a current diagnosis of only one allergic disease. By contrast, we defined allergic multimorbidity as the coexistence of 2 or 3 current allergic diseases in one individual.

### Covariates

A number of demographic characteristics and socioeconomic factors were considered covariates in the data analysis. Confounding variables included sex, school grade, obesity, residential area, perceived household economic status, paternal and maternal educational levels, and current smoking. School grade levels were grouped into “middle school” (middle school first, second, and third grades), and “high school” (high school first, second, and third grades). After calculation of body mass index (BMI) using self-reported height and body weight, obesity categories were defined as (a) underweight (< 5th percentile), (b) normal weight (5th ≤ BMI < 85th percentile), (c) overweight (85th ≤ BMI < 95th percentile), and (d) obese (BMI > 95th percentile) according to the sex- and age-specific BMI reference values presented in the 2018 Korean National Growth Chart^32^. Residential areas were categorized into three variables, namely metropolitan cities, small- and medium-sized cities, and rural areas. The information on subjective household income was assessed by asking students about their self-perceived family economic status. The answers included high, middle-high, middle, middle-low, and low. Parental educational attainment was assessed as the highest academic degree obtained. The responses were classified into four categories as follows: high school or lower, college or higher, unknown and father/mother absence. Physical activity was defined as engaging in at least 3 days of vigorous-intensity activity that made them breath hard and sweat for > 20 min a day. As a confounding variable, current smoking was defined as reporting any of following smoking experience: (a) past 30-day conventional cigarette use, (b) past 30-day electronic cigarette (e-cigarette) use, and (c) past 30-day heated tobacco product (HTP) use.

### Statistical analysis

The complex multi-stage sampling models were taken into account, and all suggested sampling weights were applied in the statistical analysis. To examine the significant differences between those with and those without SHS exposure by socioeconomic status, and the general characteristics of those with and those without lifetime and current allergic diagnosis, a chi-squared test was performed. In addition, the relationship between SHS exposure and allergic morbidity was presented as actual frequencies and weighted percentages. Logistic regression models were applied to examine the association between SHS exposure and allergic disease, controlling for the confounding variables. First, multiple logistic regression model stratified by current smoking status calculated odds ratios (ORs) and 95% confidence intervals (CIs). Next, the total sample of 55,748 were included in multinomial logistic regression model. Potential confounders introduced in the logistic regression models were selected based on prior knowledge of the literature and variables identified as being correlated with both the exposure and the outcome of interest. All statistical calculations were performed with SPSS version 26.0 (IBM, Armonk, NY, USA). The critical level of statistical significance was set at *p* < 0.05.

## Data Availability

The datasets analyzed during the current study are publicly available. Individual researchers need to gain access to download the dataset via the official website https://yhs.cdc.go.kr.

## References

[1] Asher MI (2006). Worldwide time trends in the prevalence of symptoms of asthma, allergic rhinoconjunctivitis, and eczema in childhood: ISAAC Phases One and Three repeat multicountry cross-sectional surveys. Lancet.

[2] KCDC. The Statistics of 15th Korea Youth Risk Behavior Survey(KYRBS). Korea Centers for Disease Control and Prevention, Seoul, Korea (2019).

[3] Benefits by Frequency of Disease(Total). https://kosis.kr/statHtml/statHtml.do?orgId=350&tblId=DT_35001_A074111&conn_path=I2 (2020).

[4] Kariyawasam HH, Rotiroti G (2013). Allergic rhinitis, chronic rhinosinusitis and asthma: unravelling a complex relationship. Curr. Opin. Otolaryngol. Head Neck Surg..

[5] Paller AS, Spergel JM, Mina-Osorio P, Irvine AD (2019). The atopic march and atopic multimorbidity: Many trajectories, many pathways. J. Allergy Clin. Immunol..

[6] Bousquet J, Vignola A, Demoly P (2003). Links between rhinitis and asthma. Allergy.

[7] Alduraywish SA (2017). Is there a march from early food sensitization to later childhood allergic airway disease? Results from two prospective birth cohort studies. Pediatr. Allergy Immunol..

[8] Spergel JM, Paller AS (2003). Atopic dermatitis and the atopic march. J. Allergy Clin. Immunol..

[9] France EF (2012). Multimorbidity in primary care: a systematic review of prospective cohort studies. Br J Gen Pract..

[10] Mariño-Sánchez F (2019). Multimorbidities of pediatric allergic rhinitis. Curr. Allergy Rep..

[11] Leynaert B, Neukirch C, Liard R, Bousquet J, Neukirch F (2000). Quality of life in allergic rhinitis and asthma: a population-based study of young adults. Am. J. Respir. Crit Care Med..

[12] US Department of Health and Human Services. Atlanta, GA: Department of Health and Human Services, Centers for Disease Control and Prevention, Coordinating center for Health Promotion, National Center for Chronic Disease Prevention and Health Promotion, Office on Smoking and Health. The Health Consequences of Involuntary Exposure to Tobacco Smoke: A Report of the Surgeon General (2006).

[13] WHO. Guidelines for implementation of Article 8 of the WHO FCTC. Paper presented at FCTC Conference of Parties. Geneva: World Health Organization (2007).

[14] Schick S, Glantz S (2005). Philip Morris toxicological experiments with fresh sidestream smoke: more toxic than mainstream smoke. Tob Control.

[15] Agaku IT, Singh T, Rolle I, Olalekan A-Y, King BA (2016). Prevalence and determinants of secondhand smoke exposure among middle and high school students. Pediatrics.

[16] Rumold R, Jyrala M, Diaz-Sanchez D (2001). Secondhand smoke induces allergic sensitization in mice. J. Immunol..

[17] Lanckacker EA (2013). Short cigarette smoke exposure facilitates sensitisation and asthma development in mice. Eur. Respir. J..

[18] Burgess JA (2007). Childhood allergic rhinitis predicts asthma incidence and persistence to middle age: a longitudinal study. J. Allergy Clin. Immunol..

[19] Krämer U (2004). The effect of environmental tobacco smoke on eczema and allergic sensitization in children. Br. J. Dermatol..

[20] Lee A, Lee SY, Lee K-S (2019). The use of heated tobacco products is associated with asthma, allergic rhinitis, and atopic dermatitis in Korean adolescents. Sci. Rep..

[21] Saulyte J, Regueira C, Montes-Martínez A, Khudyakov P, Takkouche B (2014). Active or passive exposure to tobacco smoking and allergic rhinitis, allergic dermatitis, and food allergy in adults and children: a systematic review and meta-analysis. PLoS Med..

[22] Burke H (2012). Prenatal and passive smoke exposure and incidence of asthma and wheeze: systematic review and meta-analysis. Pediatrics.

[23] Xie L, Atem F, Gelfand A, Bauer C, Messiah SE (2020). United States prevalence of pediatric asthma by environmental tobacco smoke exposure, 2016–2017. J. Asthma..

[24] Merianos AL, Jandarov RA, Mahabee-Gittens EM (2019). Association of secondhand smoke exposure with asthma symptoms, medication use, and healthcare utilization among asthmatic adolescents. J. Asthma..

[25] National Health Promotion Act. *Law No. 11142.* Article 9. Measures for Anti-Smoking.

[26] Tripathy JP (2020). Secondhand smoke exposure at home and public places among smokers and non-smokers in India: findings from the Global Adult Tobacco Survey 2016–17. Environ. Sci. Pollut. Res. Int..

[27] Faber T (2017). Effect of tobacco control policies on perinatal and child health: a systematic review and meta-analysis. Lancet Public Health..

[28] Frazer K (2016). Legislative smoking bans for reducing harms from secondhand smoke exposure, smoking prevalence and tobacco consumption. Cochrane Database Syst Rev..

[29] Wynne O (2018). Signs, fines and compliance officers: a systematic review of strategies for enforcing smoke-free policy. Int. J. Environ. Res. Public Health.

[30] Bayly JE, Bernat D, Porter L, Choi K (2019). Secondhand exposure to aerosols from electronic nicotine delivery systems and asthma exacerbations among youth with asthma. Chest.

[31] Kim Y (2016). Data resource profile: the Korea Youth Risk Behavior Web-based Survey (KYRBS). Int. J. Epidemiol..

[32] Kim JH (2018). The 2017 Korean National Growth Charts for children and adolescents: development, improvement, and prospects. Korean J. Pediatr..

